# Round the Hospitals

**Published:** 1920-03-27

**Authors:** 


### ROUND THE HOSPITALS.

The King held an Investiture at Buckingham Palace on March 17, and personally conferred decorations on the following members of the Nursing Services:—Royal Red Cross, Second Class.—Misses C. Bottomley, F. Child, E. Cumberlidge, L. Greany, E. Henderson, M. Pike, A. Robb, K. Rogers, M. Sinclair, and E. Slingsby, Q.A.I.M.N.S.R.; Misses H. Brotherton and M. McFall, T.F.N.S.; Miss M. Ainger, and Mrs. Annie Downing, C.N.S.; Misses E. Hackett, E. Hopwood, E. Dew-Johnson, M. Johnson, R. Warden, and Mrs. Grace Leah, B.R.C.S.; Miss A. Ensor St. John, Amb. Brig.; Misses C. Martin and H. Simpson, Civil and War Hosp.; Fede, Countess Riccardi-Cubitt, Misses E. Hague and I. Marshall, Mrs. Jeannie Sinclair, V.A.D. All these ladies were afterwards received at Marlborough House by Queen Alexandra.

**ROUND THE HOSPITALS**—(*continued*).

The King held an Investiture at Buckingham Palace on March 20 and personally conferred decorations on the following members of the nursing services:—Bar to the Royal Red Cross: Miss Marian Knox, Q.A.I.M.N.S.; Royal Red Cross, First Class: Miss Elsie Cassidy, Mrs. Margaret Fishbourne, Misses Amy Hill and Ethel James, Q.A.I.M.N.S., Miss Mable Ensor, T.F.N.S.; Royal Red Cross, Second Class: Misses R. Craddock and S. Hepworth, Q.A.I.M.N.S., Misses M. Armitage, R. Beales, M. Conway, S. Coulter, R. Dalzell, E. Fewlass, M. Fleming, A. Gamlin, L. Gibson, N. Hinch, E. Howitt, and F. Jackson, Q.A.I.M.N.S.R.; Misses E. Ellison, and M. Hughes, Mrs. A. Fellowes, Mrs. L. Johnson, Mrs. E. Poyntz, T.F.N.S.; Misses E. Armitage, K. Freer, K. Holmes-Hardwicke, A. Hotchkiss, L. Howarth, and M. Stratton, C.N.S.; Misses L. Bale, G. Bower, J. Boyd, J. Brown, M. Foreman, E. Goodall, L. Griffiths, K. Hallett, and A. Lyon, B.R.C.S.; Miss E. Newitt, St. John Ambulance Brigade; Misses B. Dudley and E. Edwards, Civil and War Hospital; Misses E. Arnott, M. Baxendale, A. Belling, E. Carter, A. Cooper, C. Dalton, M. Frost, H. Forster, Mrs. M. Eltringham, Mrs. E. Fry, Mrs. J. Hartnett, Mrs. J. Hopkins, and Mrs. A. Isaac, V.A.D. Queen Alexandra received the above ladies together with Miss A. B. Smith, R.R.C., at Marlborough House after the Investiture.

Many fine things were said in the newspapers about Nurse Cavell and the profession to which she belongs on the day succeeding the unveiling of her memorial by Queen Alexandra. The occasion was hailed as a national event, and Her Majesty's words struck the note which has re-echoed since throughout the Empire: "The countless thousands who will pass this spot in our time and in future generations will think with sorrow of her cruel death, with pride of her splendid fortitude, and with affection of her unselfish and womanly character." Countless crowds visited the memorial through the week, and many laid tributes of flowers at the base. No comment on the occasion is more wholesome than that contributed by a writer in the *Globe*, which we would gladly give in full had we space: "There was never a time when we had greater need to lay to heart the lessons she taught us by her life and death. . . . In unity and in fortitude we shall find the remedy for all our present ills, and it is not for the countrymen of Edith Cavell to shrink from the plain duty which is laid upon us to-day." All who love their country, all who honour the nursing profession must feel touched with gratitude to Viscount Burnham and his coadjutors that so great an object-lesson in patriotism, fortitude, and Christian conduct should have been afforded London in this beautiful memorial. The Nurses' Shilling Fund goes steadily mounting up and has now reached a total of 218,437.

One of Edith Cavell's favourite books was the "Meditations of Thomas à Kempis." She had her copy with her in prison and marked favourite passages, some of which have a very poignant bearing on her circumstances. On the fly-leaf she wrote a brief pathetic summary of her arrest, imprisonment, and sentence. Mr. Humphrey Milford (Oxford University Press) is publishing a facsimile of this little volume reproducing the private marks; it is called the Edith Cavell Edition, and will be sold at a price of from 2s. 6d. upwards, according to binding, for the benefit of the Edith Cavell Homes of Rest for Nurses. It contains an interesting introduction by Bishop Ryle, Dean of Westminster, and one passage marked with the date "St. Gilles, 11 Oct.," the day on which she received her sentence, and only a few hours before her execution, runs: "There is none to help me, none to deliver and save me, but Thou O Lord God, my Saviour, to whom I commit myself and all that is mine, that Thou mayest keep watch over me and bring me safe to life everlasting."

There is evidence of the existence in Germany of a strong body of opinion which unreservedly condemns the execution of Edith Cavell and other acts of brutality committed in Belgium by the German military governors during the occupation. The Central Soldiers' Council urged the appointment of a high court of justice to inquire, among other things, into the trial of Miss Cavell, and demand the production of the documents in the case. This Council named General von Sauberzweig as the principal person to be indicted. He was German Military Commandant of Brussels at the time of her arrest, and it was he who signed her death-warrant. It now transpires that his orders were "to make an example"; "to frighten those who might presume on their sex to take part in enterprises punishable by death." All the details of the proceedings prior to the arrest of Miss Cavell show conclusively her insight in refusing to make any personal appeal to the judge. From the beginning she realised that she was to be "an example," both on account of her sex and nationality. The trial was a sham. Those who wish to study the new documents at further length will find them very clearly outlined in *The Times*, March 8 and 9.

A writer in the *Daily Chronicle* calls attention to the fact that, with the exception of Royalties, the only public statues of women erected in this country are of Florence Nightingale, Edith Cavell, "Sister Dora" (in Walsall), and Mrs. Siddons. The proportion is instructive—three nurses to one actress. It is strange that no woman author, professor or doctor should have been honoured in this way. At Bremhill, in Wiltshire, however, there is a statue of Maud Heath, a peasant woman who lived in the reign of Richard II. and was noted for her beneficent deeds.

Sir Alan Garrett-Anderson has presented to the Elizabeth Garrett-Anderson Hospital, in the Euston Road, an adjoining house, which will provide the

**ROUND THE HOSPITALS**—(*continued*).

nursing staff with the means of expansion, and, we trust, with more cheerful quarters than are at present available for them. Lady Lytton, presiding at the annual meeting of the governors, stated that the Matron hopes, by means of this increased accommodation, to arrange an eight-hour day for the nurses. The hospital, being exclusively for women and children, is not in a position to give a complete course to its probationers, but co-operates with institutions like the Dreadnought at Greenwich, which furnish only training in male medical and surgical wards.

The annual meeting of the Victorian Bush Nursing Association was held at Federal Government House (Melbourne) on September 24, Her Excellency, Lady Helen Munro Ferguson, presiding for the last time, owing to her approaching departure from Australia. Proceedings disclosed that arrangements had been made whereby a nurse was to be sent to Manungatang, the soldiers' settlement, and that for the first year they were to have her services free. The means whereby this has been made possible have come from the State War Council and the Macpherson Trust Fund. Could this be done at all soldiers' settlements it would very materially assist the repatriation process. A country representative narrated the interesting fact that his committee was considering the possibility of establishing a cottage hospital in connection with the Bush nurse's work. The committee had already decided to build a house for the nurse, and it had been proposed that a small hospital might also be provided. But the narrator failed to remark that here at once was work for two women—one for nursing and one for housework and cooking.

After a discussion on the question of salaries in New South Wales (the Bush nurse is sometimes paid £170 per annum, and never less than £150, salaries which have drawn away not a few Victorians, it was resolved to raise the present salary of £135 per annum to £150, and to let the Bush nurse have the privilege of deciding upon her residence. Discussion also brought out the fact that whenever a doctor settles in a district a tendency to dispense with the nurse's services manifests itself. This should be stoutly resisted.

A full inquiry was held on March 11 by the Visiting Committee of the Whitchurch (Cardiff) Mental Hospital into the charges brought against the conduct of the nursing during the war under the medical superintendent, Colonel Goodall. The matron, deputy matron, sisters, staff nurses and others attended, and the conclusion arrived at was that the statements made by certain male attendants to the Cardiff Trades and Labour Council relating to moral and physical risks run by the female nurses had been greatly exaggerated and are unjustified. The committee was fully convinced of the superior advantages of nursing by women in the interests of patients. The people who made these allegations refused to give evidence and made no attempt to substantiate their charges. A memorial was put in signed by thirty-one ward sisters, staff nurses, and senior probationers denying the sensational stories which have been circulated. The evidence given went dead against the contentions of the disaffected male attendants. During the 1917-19 period of the military occupation, when the 465 mental beds were occupied by soldiers, only twelve nurses were struck by patients and three injured, whereas in the civil period 1914-15 forty-seven nurses were struck and seventeen injured by the women patients. The inquiry was the result of a campaign to get the male wards staffed by male nurses, and the result entirely vindicates the new system.

Mr. and Mrs. Martin Harvey gave a fine performance at the Lyceum Theatre, in Edinburgh, on March 11 in aid of the beautiful little cottage home at Shanklin for nurses disabled in the war. The play was Maeterlinck's "Burgomaster of Stilemonde." Between the acts Mrs. Martin Harvey pleaded the cause of the "wonderful and gallant nurses" who are now suffering in consequence of their courageous deeds during the war. People, she said, ought to know what they owed to these "magnificent heroines." When they were healed and returned to their work again the home would be to them a house of rest. At present they needed such homes and all the help they could get.

The Hon. Sir Arthur Stanley expressed his satisfaction on his return from the recent meetings of the General Council of the League of Red Cross Societies at Geneva at the line of action adopted. The Conference was largely occupied with defining the position of the newly-formed League of Red Cross Societies as related to the existing International Red Cross Committee, sitting at Geneva. It is intended that the two institutions shall work together with cordial understanding and mutual confidence. The admission of the Central Powers into the League came under discussion, but was postponed until the full board of governors be constituted. The League of Red Cross Societies is the outcome of the Covenant of the League of Nations. It represents twenty-seven nations, and its work falls naturally into two distinct sections, administrative and medical.

The Imperial Nurses' Club (137 Ebury Street, S.W. 1) is appealing for more subscriptions. It is in no sense a charity, but it is a very valuable institution and as such is worthy of aid in the gallant struggle to meet expenses at this exceptionally costly time. Last year it proved its ability to meet all the ordinary outgoings out of members' subscriptions and payments. But, as all club committees know, this does not mean that alterations and extensions can be met out of current income. On the occasion of the Queen's surprise visit on March 13 Her Majesty was informed that since the Club was opened three years ago nearly 14,000 nurses had

**ROUND THE HOSPITALS**—(*continued*).

been accommodated under its roof for varying periods. Few of the members are in a position to lay down capital sums for the extensions so urgently required, and subscriptions for this purpose will be gladly received by the Hon. Treasurer, Colonel W. McAdam Eccles, 124 Harley Street, W. 1.

---

THE retirement of Miss Eleanor Barton from the position as Matron of Chelsea Infirmary, which she has held for so many years, can only not be reckoned a calamity for the nursing world by the promise it holds that her activities will be turned wholly to the higher organisation of the profession. Miss Barton was one of the earlier matrons to recognise that her responsibilities as a nurse did not begin and end with the institution in which she was called to rule. She acquired the habit of lucid speech, gave much concentrated attention to matters relating to the profession at large, and however busy did not deny her personal co-operation in council when occasion called her. By evident tokens this broad policy was seen to react for good upon her own training-school, which has risen to a level of which her Board are justly proud. The war, during which she was Principal Matron of the Third London General (Territorial) Hospital, a position involving an immense output of energy, taxed her strength severely. We believe she is well inspired in relinquishing her present post before the continuous strain, physical and mental, have lessened her unusual powers.

---

IT is still very rare for women to be selected for that peculiarly English compliment, the bestowal of the "freedom" of the city or borough. All the more reason is there to be proud of the honour which the West Bromwich County Council are about to confer on Dame Sidney Browne, R.R.C., in making her a burgess of the borough. Dame Sidney Browne was for thirty years from babyhood a resident in the town, and served on the staff of the West Bromwich District Hospital. In making her a burgess the Council show their recognition of the work accomplished during the war by the Service she represents.

---

THE three best probationers of the year at King Edward VII. Hospital, Cardiff, are Nurse Martha E. Prothero, Nurse Gladys M. Davies, and Nurse Margaret O. Jones. They were presented in the order named with gold, silver, and bronze medals respectively by Lady William James Thomas on March 10. Dr. Patterson, in commending the work of the nurses, stated that the course of training had been extended from three to four years, and that the standard required was higher than ever. He was also able to state that the demand for nursing training was increasing every year. This bears out our own opinion that the shortage of suitable candidates is purely local and temporary. In many places it has already ceased to be felt. In some it has never appeared.

SOUTHAMPTON is one of the places where probationers are still scarce. Notwithstanding great improvements at the Shirley Warren Infirmary under the progressive Board of Guardians at Southampton, some reforms have still to be delayed owing to a difficulty in getting sufficient nurses. The chairman is Mrs. Palmer, who has a considerable knowledge of nursing requirements, and has done much to raise the standard of comfort and well-being for all the nurses under the control of the Board. She claims that the rate of pay is higher than that of any similar institution in the country. A 49-hour week has been instituted, but a certain amount of overtime work, paid for at overtime rates, has to be tolerated until the staff is brought up to full strength.

---

OWING to the difficulty in getting probationers willing to bind themselves for a three-year course of fever training the Metropolitan Asylums Board proposes to revert to the two-year system. The principal medical officer has been in consultation with matrons from the various fever institutions, and reports that the majority are in favour of this change. He recommends that probationers should now sign on for two years only; but that the alteration should at first be tentative, and that its effect on the service should be watched. The result might be to increase the number of the probationers at the expense of the fully-trained staff nurses. He also recommends that assistant nurses, Class II., should no longer be a recognised grade at the acute hospitals. When temporary help is required, assistant nurses should be engaged, and if later on they prove satisfactory and desire to join the permanent staff, they are to be allowed to count six months of temporary work (instead of three as at present) towards their period of training as probationers.

---

FOUR midwives were struck off the roll at the recent meeting of the Central Midwives Board for Scotland, which took place at 49 Lauriston Place, Edinburgh, with Sir J. Halliday Croom in the chair. The penal cases were such as to illustrate very forcibly the necessity in the interest of Scottish mothers for the board, whose protective operations are only at the commencement. One of the midwives deprived of their calling had recently been convicted at Perth of keeping an improper house, and had been fined 40s., with the alternative of twenty days' imprisonment. Another of those struck off the roll had been sentenced to six months' imprisonment for theft and fraud. A third had been sentenced to eighteen months' imprisonment for using instruments with intent to procure abortion. And the fourth was proved to have been in contact with a patient suffering from puerperal fever without making notification or taking the required precautions. One midwife was suspended for three months for failure to notify a case of ophthalmia neonatorum, whereby the child's eyes were permanently impaired.

